# Willingness to pay for osteoporosis risk assessment in primary dental care

**DOI:** 10.1186/s13561-019-0232-z

**Published:** 2019-05-24

**Authors:** Helena Christell, Joanna Gullberg, Kenneth Nilsson, Sofia Heidari Olofsson, Christina Lindh, Thomas Davidson

**Affiliations:** 1 Faculty of Odontology, Malmö University, Box 50500, 202 50 Malmö, Sweden; 2 Department of Radiology, Helsingborg Hospital, 251 87 Helsingborg, Sweden; 3 Centre for Medical Technology Assessment, Department of Medical and Health Sciences, Linköping University, 581 83 Linköping, Linköping, Sweden

**Keywords:** Dental clinics, Health economics, Medico economic, Osteoporosis, Patient acceptance of health care, willingness to pay, I10

## Abstract

**Background:**

Fragility fracture related to osteoporosis among postmenopausal women is a significant cause of morbidity. The care and aftercare of these fractures are associated with substantial costs to society. A main problem is that many individuals suffer from osteoporosis without knowing it before a fracture happens. Dentists may have an important role in early identification of individuals with osteoporosis by assessment of dental radiographs already included in the dental examination. The aim of this study was therefore to investigate postmenopausal women’s preferences for an osteoporosis risk assessment in primary dental care.

**Results:**

Most respondents (129 of 144 (90%)) were willing to pay for an osteoporosis risk assessment in primary dental care. The overall mean willingness to pay (WTP) including respondents that denoted none or zero WTP was 44.60 € (CI 95% 38.46–50.74 €) (median 34.75 €). A majority (80.6%) of the respondents that denoted WTP also gave a motivation for their answer. The two most common reasons denoted for being willing to pay for osteoporosis risk assessment were the importance of early diagnosis and preventive care to avoid fractures (41.0%) and the importance of knowledge of a risk of osteoporosis (26.4%). A majority of respondents (67.8%) considered it valuable if dental clinics would offer osteoporosis risk assessment.

**Conclusions:**

Postmenopausal women seem to find it valuable to be offered osteoporosis risk assessment in primary dental care and are willing to pay for such a risk assessment. From a societal perspective early diagnosis of osteoporosis by risk assessment in primary dental care could prevent osteoporotic related fractures and benefit women’s health and quality of life, as well as have a major impact on the health-care budget in terms of cost-savings.

## Introduction

Osteoporosis is a metabolic disease characterised by progressive reduction in bone mass and changes in the micro architectural structure of bone. The disease leads to an increased risk of fractures, most commonly affecting the spine, forearm, and hip [1]. At the age of 50, the lifetime risk of one of these fractures is 22% and 46% in men and women, respectively [2]. The total cost burden of osteoporosis and its related fractures in Europe was estimated to 30.7 billion euros in 2010 and expected to increase to 38.5 billion euros by 2025 [3]. Consequently, osteoporosis is a major health problem that imposes a growing financial burden on health services and the burden becomes even greater when taking the Quality Adjusted Life Years (QALYs) lost into account [2].

Osteoporosis is diagnosed based on bone mineral density (BMD) derived from Dual-energy-X-ray Absorptiometry (DXA) [4]. Although there are risk factors other than low BMD, a major challenge in managing osteoporosis is the difficulty in identifying affected individuals before the condition is established and fracture has occurred [4–6]. Research has shown that assessment of bone tissue in radiographs obtained at dental clinics may disclose findings that constitute a risk factor for skeletal fracture [7]. Dental radiographs are probably the most commonly performed radiographic examination in the world [8]. These radiographs show the teeth as well as a varying amount of bone tissue which makes them a potential diagnostic tool for early identification of risk indicators of osteoporosis and consequently of fractures. Extended use of dental radiographs for risk assessment of osteoporosis, could make dentists possible ‘gate-keepers’ of bone health and contribute to reduce the huge socioeconomic burden that this condition means.

Dentists’ attitudes towards chairside medical screening has been investigated and it was found that dentists considered medical screening important and were willing to implement this into their practice [9, 10]. Results from other studies indicate that a majority of patients were willing to undergo medical screening in a dental setting as they considered this important [11, 12]. Gullberg et al. 2018 [13] investigated the attitudes of Swedish dentists, patients, and medical specialists towards osteoporosis risk assessment in primary dental care using qualitative study design. Several barriers at the individual level and within the healthcare system were identified as possible obstacles to optimal implementation of osteoporosis risk assessment at dental clinics. One of these barriers was the extra time, and thus additional cost, required for supplementary assessment of dental radiographs.

All interventions in health care should be deemed cost-effective in order to effectively use the scarce resources. For this reason, health economic evaluations are used, most commonly for pharmaceuticals, but also for diagnostic procedures and medical devices [14]. Cost benefit analysis is one type of economic evaluation that enables a direct answer of whether the benefits of a method exceeds its costs, thus having a positive net social benefit, indicating that the method is worthwhile. The benefits of any method for screening, diagnosis or treatment could be estimated in terms of willingness to pay (WTP) [15], using contingent valuation. In the context of osteoporosis risk assessment in a primary dental care setting WTP would reveal the strength of individual preferences for this method to be applied. According to our knowledge there are no previous studies addressing WTP of osteoporosis risk assessment in primary dental care.

The aim of this study was therefore to investigate postmenopausal women’s preferences for an osteoporosis risk assessment in primary dental care using a self-administered questionnaire to assess WTP.

## Material and methods

Data was collected between May and August 2013 in two primary dental clinics in southern Sweden, one public clinic and one private. Female patients over 50 years of age that could read and comprehend Swedish were consecutively invited to participate.

### Information booklet and questionnaire

A booklet (Appendix I) was produced containing information about osteoporosis, its risk factors, prevalence, and possible consequences. The scenario of identifying women with osteoporosis risk was illustrated in two steps with i) risk assessment of women over 50 years based on current dental radiographs at the dental clinic and the possible diagnostic accuracy for such a method and ii) the procedure of referring women at risk of osteoporosis for further investigation with DXA.

A questionnaire (Appendix II) was developed in consultation with a health economist. The first section of the questionnaire aimed to measure the respondents’ WTP for an osteoporosis risk assessment at the dental clinic using a payment scale. To enhance the probability of receiving a true value of the WTP the respondents were asked to denote the maximum amount that they were willing to pay as well as to cross out the amounts that exceeded this. The WTP question was followed by an open-ended question where the respondents were asked to write down a motivation for their answer to the first question. The respondents were also asked if they considered it valuable to be offered an osteoporosis risk assessment in connection with a dental visit. The dental staff was instructed to consecutively distribute the questionnaires and the information booklet to female patients in association with their dental visit and to encourage the patients to read the information booklet before completing the questionnaire. The information booklet was only distributed to the patients at the public clinic.

### Focus group

Prior to distribution of the questionnaire to the dental clinics, a focus group interview was performed with participation of four postmenopausal women, between 50 and 84 years of age. The women were asked to read the information booklet and answer the questionnaire. During the subsequent discussion they were asked to give feedback regarding their understanding and interpretation of the booklet and survey questions. A minor revision of the questionnaire and booklet was then made according to the results from the focus group interview.

### Statistical calculations

For statistical calculations IBM SPSS statistics 24 software for Windows (SPSS Corporation, Chicago, Illinois, USA) was used. The data were subjected to descriptive statistics including 95% confidence intervals (CI). Correlation between mean WTP and any of the variables was analysed with one-way ANOVA. *P* < 0.05 was considered statistically significant. The dependent variable was willingness to pay for osteoporosis risk assessment in primary dental care and the independent variables were: osteoporosis diagnosis, osteoporotic fracture, a relative with a diagnosis of osteoporosis or osteoporotic fracture, age, educational level, occupation, and annual income.

The study followed the ethical considerations of the Declaration of Helsinki according to which no ethical approval was necessary as participation in the interviews was voluntary, anonymous and no data can be traced back to the respondents. Information about the study, formulated according to the general outlines provided by the Regional Ethical Review Board, Lund, Sweden was provided in the information booklet administered together with the questionnaire. The costs were based on cost-year 2013 when one euro averaged 8.6 SEK.

## Results

**Fig. 1:**
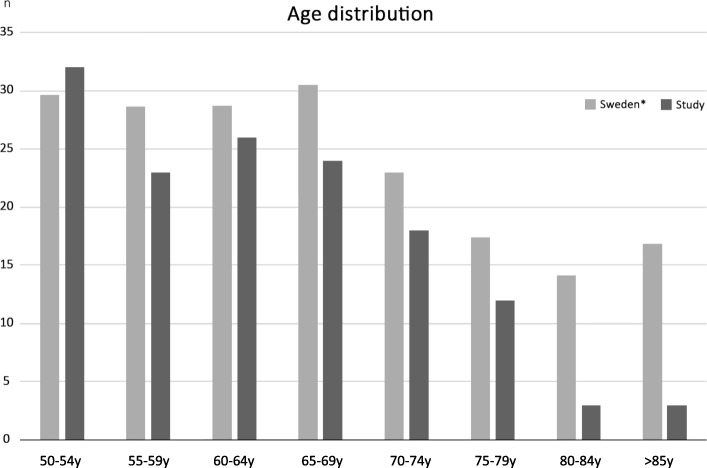
Correspondence between the age distribution in the study population compared with that in the Swedish population of women in different age groups between 50 and 85+ (*times 10,000) [16]

In total 144 women answered the questionnaire. Table 1 presents demographic characteristics of the respondents. Figure 1 illustrates how the age distribution of the eight age groups between 50 and 85+ correspond to the population of Sweden 2013 [16].

**Table 1:** Demographic characteristics of the respondents (in total *n* = 144)

		n
Age (years)	50–54	32
	55–59	23
	60–64	26
	65–69	24
	70–74	18
	75–79	12
	80–84	3
	> 85	3
	No answer	3
Education	Primary	34
	Secondary	39
	College	68
	No answer	3
Occupation	Full time	46
	Part time	34
	Retired	59
	Student	1
	Unemployed	0
	Other	1
	No answer	3
Annual income (Euro)	0–22,999	40
	23,000–34,999	34
	35,000–45,999	39
	46,000–57,999	8
	> 58,000	3
	No answer	20
Osteoporosis diagnosis	Yes	19
	No	121
	Do not know	1
	No answer	3

**Table 2:** Number of respondents (*n* = 141 of 144 due to no answer of WTP for three respondents) divided into different groups of annual income and median willingness to pay (WTP), mean WTP and 95% CI of mean WTP in Euro (€).

Annual income	*n* (total *n* = 141)	Median WTP	Mean WTP	CI 95% of mean WTP
0–22,999	39	34.75	36.49	27.22–45.87
23,000–34,999	33	34.75	48.77	34.40–63.25
35,000–45,999	39	57.92	53.17	39.39–66.96
46,000–57,999	8	46.34	53.63	16.22–90.94
> 58,000	3	34.75	54.10	- 81.90 – 190.10
No answer of income	19	23.17	31.05	20.39–41.82
Overall results		34.75	44.60	38.46–50.74

### Willingness to pay

Most respondents (129 of 144 (90%)) were willing to pay for an osteoporosis risk assessment in primary dental care. The overall mean WTP including respondents that denoted none or zero WTP was 44.60 € (CI 95% 38.46–50.74 €) (median 34.75 €) (Table 2). A majority (80.6%) of the respondents that denoted WTP also gave a motivation for their answer. Twelve respondents denoted WTP zero and three answers regarding WTP were missing. The two most common reasons denoted for being willing to pay for osteoporosis risk assessment were the importance of early diagnosis and preventive care to avoid fractures (41.0%) and the importance of knowledge of a risk of osteoporosis (26.4%). A majority of respondents (67.8%) considered it valuable if dental clinics could offer osteoporosis risk assessment.

There was no statistically significant association between income and WTP, thus no income elasticity (Table 2). None of the variables age, education level, occupation, osteoporosis diagnosis, osteoporotic fracture, having a relative with osteoporosis diagnosis or osteoporotic fracture, showed any statistically significant influence on the respondents’ WTP when controlled using one-way ANOVA (Table 3). Mean WTP was slightly lower for those who already had an osteoporosis diagnosis or had suffered from an osteoporosis-related fracture, while it was somewhat higher for those who had relatives diagnosed with osteoporosis or had suffered from an osteoporosis-related fracture. The lower WTP from respondents that had already been diagnosed with osteoporosis was in most cases motivated in the answer by the fact that there was no need for diagnosis. Most respondents diagnosed with osteoporosis and/ or with relatives diagnosed with osteoporosis that denoted high WTP stated the importance of early diagnosis.

**Table 3:** The number of respondents that answered the question if they had been diagnosed with osteoporosis, had an osteoporotic fracture, had a relative with osteoporosis or a relative that had an osteoporotic fracture related to their denoted willingness to pay (WTP) (*n* = 141 of 144 due to no answer of WTP for three respondents) (*P* > 0.05) in Euro (€)

	Diagnosed with osteoporosis		Osteoporotic fracture		Relative with osteoporosis		Relative with osteoporotic fracture	
	*n*	mean WTP	*n*	mean WTP	*n*	mean WTP	*n*	mean WTP
Yes	17	43.62	7	39.73	15	48.67	10	57.94
No	120	45.00	131	45.20	106	43.40	111	42.38
Do not know	1	57.92	2	39.62	18	49.58	19	52.48
No answer of these questions	3	30.93	1	11.58	2	34.75	1	11.58
ANOVA Sig. level		0.932		0.919		0.741		0.284

50-54y 55-59y 60-64y 65-69y 70-74y 75-79y 80-84y >85y

### Net social benefit

The benefits related to the costs can be expressed as the net social benefit (NSB) [15, 17]. NSB for osteoporosis risk assessment in primary dental care can be calculated as the WTP (a) minus the cost for the dentist’s working time (b). The expected revenue per hour in 2013 for a general dentist was around 220 € (3.67 € per minute) and the working time for a dentist to perform an osteoporosis risk assessment is estimated to be ten minutes. The average WTP (a) of 44.60 € minus a cost for ten minutes of a dentists working-time (b) of 36.68 € would result in a positive NSB. Any patient-related cost for transport to the clinic and the time to get to the clinic can be estimated to zero as this is already included in the cost for the dental visit and the cost of the patient’s time for the actual risk assessment can be assumed to be negligible.

There is a well-known weakness in cost-benefit analyses in that the respondents may denote a higher WTP than the amount of money they would pay for an intervention in real life. Hence there is a risk that the resulting value of WTP is overestimated. The patient-fee for visiting a medical specialist in 2013 in Sweden, thus the cost a patient would normally have to pay to get an assessment of osteoporosis, was around 25% lower than the estimated average WTP in this study. In countries where medical care is paid for by the patients themselves or by private insurances the WTP for such risk assessment would be expected to be higher. With a 25% lower value for WTP, however, risk assessment of osteoporosis in primary dental care would still be resources well-invested in a long-term perspective as the benefits from early detection of osteoporosis would include reduced future health-care costs for osteoporotic-related fractures.

## Discussion

The results showed that most respondents denoted a WTP indicating that women over 50 years of age are willing to pay for osteoporosis risk assessment and find it valuable to be offered this at dental clinics. The average WTP overrated the estimated cost for osteoporosis risk assessment meaning that there is a positive net social benefit indicating that the method for risk assessment is worthwhile. This agrees with the results of another study where a majority of women aged 60 and older indicated preference for osteoporosis screening [18]. Only women over 50 years of age were included because postmenopausal women are at an increased risk of osteoporosis and this selection of respondents that have been diagnosed with the examined disease is in line with most other studies regarding WTP for diagnostic technologies [19]. The number of respondents was comparable with that in other WTP-studies in dentistry ranging from 36 to 611 respondents [17, 20–23].

WTP has proven to be influenced by study design and elicitation methods [19]. Studies using face-to-face interviews allow for communication with the respondents, which can avoid misunderstandings of the questions resulting in a higher validity than studies based on self-administered questionnaires [24, 25]. Payment scale implies a risk that the respondents are guided by the range of bid and many respondents tend to choose a higher value when the payment scale is long, i.e. including higher values [25]. According to two recent systematic reviews, face-to-face interviews were used in a majority of studies of WTP in oral health care [26] while most WTP-studies of diagnostic methods in medical care used self-administered questionnaires [19]. In present study a self-administered questionnaire with closed-ended questions and a payment-scale to elicit the WTP-question was constructed according to examples found in the literature [27]. The response rate was excellent with all respondents but three completing the WTP-question. According to Donaldson et al. (1997) using payment scale rather than an open-ended approach increases the likelihood that the WTP-question will be answered [27]. Furthermore, it is less time-consuming to use self-administered questionnaires instead of face-to face interviews thus enabling a larger study sample and minimising biased WTP-answers due to influence from an interviewer.

Describing a realistic scenario is fundamental in WTP-studies as it makes the respondents feel bound to their denoted value for WTP and that this value is in level with their budget [24] but it is one of the main difficulties in contingent valuation [15]. A realistic scenario when evaluating a method for diagnosis or risk assessment includes information of health gains, duration, diagnostic accuracy and risks. Such information regarding osteoporosis risk assessment was included in the booklet that was distributed to the respondents in the public clinic. The scenarios in WTP-studies often mean that the patients are asked if they are willing to pay for an intervention that is not (yet) available (a medicine or treatment that is being researched) or for medical healthcare that is normally paid for by taxes or included in the medical insurance. This could make the scenario difficult to understand which may lead to opposition of the survey in terms of a protest answer. Hence, the respondents denote a WTP that equals zero or is unreasonably high which obstructs the possibility of proper estimation of the WTP. All 15 respondents that denoted zero or non-response also motivated their answers, which were interpreted to reflect a true WTP. The WTP-method has been criticised for placing monetary value on health benefits or the saving of a life. For most patients in dental care, however, the WTP-scenario would seem realistic as they are used to being confronted with cost proposals for different treatments with specific health benefits. Furthermore, as opposed to medical care, the lion’s share of dental care is paid for by the patients and this makes the WTP-instrument applicable for valuation of the benefits of any intervention in dentistry.

In contingent valuation the optimal validation would be to compare the estimated WTP with the corresponding value of a reference method. In present study, it would mean a comparison of the measured WTP with the amount of money women over 50 years of age would really pay for an osteoporosis risk assessment in primary dental care. In most cases such comparisons are not possible as the intervention being investigated is not (yet) used in healthcare or dental care. It is recommended that one controls the measured WTP with higher income, education, disease severity, family history, and a more accurate test as WTP increases with these variables. This is a well-known weakness in contingent valuation [15]. Most studies of WTP of diagnostic methods report correlation, significant or non-significant, between WTP and the variables above [19]. The systematic review of WTP of methods in dental care found that income elasticity was tested in only 11 of 26 studies whereof eight reported a statistic correlation between WTP and income and three studies showed a tendency for higher WTP at higher income [24].

In the present study, there was an association between income level and WTP but with no statistical significance. This result is similar to several other studies of costs related to benefits in terms of WTP in dental care [17, 22, 23]. Most patients in the private clinic were over 65 years old and retired whilst most of the respondents in the public clinic were in the age range 50–64 and still working. Despite this, the respondents in the private clinic denoted a slightly higher mean WTP compared with respondents in the public clinic (not statistically significant). It is possible that some of the respondents who stated a high WTP also had real estate or bank savings and that inclusion of a question about other assets than income would have resulted in income elasticity. Furthermore, the literature background information on the disease and method for diagnosis as well as on the WTP-instrument is considered important when estimating WTP [15, 24]. Due to a misunderstanding by the staff in the private clinic, these respondents did not receive such information and it is possible that had they been given such information it would have resulted in a higher mean WTP for the respondents in the private clinic. Nineteen respondents had previously been diagnosed with osteoporosis whereof 16 denoted WTP. According to the results of other studies, there is no clear correlation between WTP and earlier diagnosis [19]. Nevertheless, a majority (13 of 19) of these respondents motivated the denoted WTP in a way that indicated that they had a positive attitude towards osteoporosis risk assessment at dental clinics whether or not they had specified any WTP. Another way to validate the estimated WTP was to have a follow-up question where respondents were asked to motivate their stated WTP, and almost all respondents gave a motivation that was logical and thus corresponding to the level of WTP. The respondents who had already been diagnosed with osteoporosis denoted either relatively low or relatively high values for WTP. The most common motivation for a low WTP was that the respondent personally would not benefit from a risk assessment of osteoporosis. A high WTP was denoted with the motivation that it was important for (other) women to be given the opportunity for this type of risk assessment of osteoporosis as early detection leads to less severe consequences.

According to a systematic review of WTP of diagnostic methods from 2013, most studies reported a median WTP below $ 100 [19]. The median WTP of 34.75 € in the present study correspond well to the WTP in other studies of screening of osteoporosis [19]. Furthermore, the patient-cost for a specialist in medical care in 2013 in southern Sweden was 35 €, thus the cost that the patients expect to pay. To enhance the probability of a realistic WTP the respondents were asked to mark the amount they were willing to pay and to cross out all the amounts exceeding their WTP but few respondents completed this question. Nevertheless, most respondents denoted a logical explanation for their WTP, which indicates a high validity. Most publications of contingent valuations focus on validation of the WTP-instrument instead of performing a full evaluation [15]. This might be due to inherent difficulties in measuring WTP and the ongoing debate concerning different ways of asking the questions and who should ask them. Incorporating WTP-studies into economic evaluation, however, requires identification of the opportunity cost of the new intervention [15]. Currently, there is no risk assessment for osteoporosis thus it would be a complex task to estimate the opportunity cost and the health benefits foregone for using this screening method. Consequently, the measured value for WTP can be assumed to represent the incremental value of risk assessment for osteoporosis compared with no risk assessment as the value of the latter could be considered non-existent. The result of a positive NSB where the estimated WTP was higher than the estimated costs indicates that risk assessment of osteoporosis is worthwhile. For comparison, a model study that assessed screening using DXA with subsequent hormone replacement concluded that DXA and treatment with Alendronate was the most cost-effective approach with $ 55,000 per QALY [28]. Another model study concluded that the most cost-effective screening strategy would be to use DXA − 2.5 with re-screening every five years for a cost of less than $50,000 per QALY [29].

## Conclusions

A majority of postmenopausal women find osteoporosis risk assessment in primary dental care valuable and are also willing to pay for such a risk assessment. Their main reasons for being willing to pay are the importance of an early diagnosis and preventive care to avoid fractures. To ensure that risk patients receive adequate treatment, dentists’ preferences of conducting osteoporosis risk assessments as well as the referral pathways and communication between primary dental care and general healthcare should be assessed.

Osteoporosis risk assessment in primary dental care could identify disease at an early stage and avoid osteoporotic fractures, which would free resources that are currently designated to treatment and rehabilitation. Future research should further assess benefits in terms of health and cost of examinations in primary dental care lying beyond the oral cavity in order to increase knowledge of extended collaboration with the general healthcare.

## Abbreviations

BMD: Bone mineral density
DXA: Dual energy x-ray absorptiometry
NSB: Net social benefit
QALY: Quality adjusted life years
WTP: Willingness to pay
